# USE OF FDA-APPROVED ALZHEIMER’S DISEASE MEDICATIONS AMONG NON-INSTITUTIONALIZED U.S. ADULTS AGE 60 YEARS AND OLDER

**DOI:** 10.1093/geroni/igz038.1737

**Published:** 2019-11-08

**Authors:** Steven Frenk, Jessica M Sautter

**Affiliations:** 1 CDC/National Center for Health Statistics, Hyattsville, Maryland, United States; 2 University of the Sciences, Philadelphia, Pennsylvania, United States

## Abstract

This study examined the use of Alzheimer’s Disease medications (ADMs) among non-institutionalized U.S. adults aged 60 years and older. It described the characteristics of ADM users, compared them to adults who were not currently using ADMs, and examined how demographic characteristics, living arrangements, healthcare utilization, and self-perceived cognition are associated with use of ADMs. It also identified the duration of ADM use and the most commonly used ADMs. Data come from the National Health and Nutrition Examination Survey, a nationally representative study of the non-institutionalized U.S. population (2009-2014). Respondents were classified as ADM users if they reported using one or more of the following prescription ADMs in the past 30 days: donepezil, rivastigmine, memantine, galantamine, or a drug combination of donepezil and memantine. During the study period, 1.9% of non-institutionalized U.S. adults aged 60 years and older reported using an ADM in the past 30 days. Use of ADMs was associated with concurrent use of antidepressant medications and also frequent or worsening memory loss and confusion. Most ADM users (82.3%) reported using ADMs for more than one year and the most frequently used ADM was donepezil. In 2009-2014, about 1.1 million non-institutionalized adults aged 60 years and older used an ADM in the past 30 days. They reported relatively poor cognitive abilities, frequent healthcare utilization, and concurrent use of antidepressant medications compared to non-users. Newly released public survey data will be useful to provide updated estimates of ADM use in the community.

